# Children or adolescents who lost someone close during the Southeast Asia tsunami 2004 – The life as young

**DOI:** 10.1002/brb3.2563

**Published:** 2022-03-25

**Authors:** Petra Adebäck, Lena Lundh, Doris Nilsson

**Affiliations:** ^1^ Division of Family Medicine and Primary Care Department of Neurobiology Care Sciences and Society Karolinska Institutet Stockholm Sweden; ^2^ Academic Primary Health Care Centre Region Stockholm Sweden; ^3^ Department of Behavioral Sciences and Learning University Linköping Sweden

**Keywords:** adolescents, children, loss, mix‐method, natural disaster, trauma

## Abstract

**Introduction:**

To lose a person close suddenly, during childhood or adolescence, can be devastating. Many children or adolescents experienced the 2004 Indonesian tsunami when they were between 10‐ and 15‐years‐old. This study, from Stockholm, Sweden, describes the long‐term effects of loss, eight‐ or nine‐years post disaster, in young adulthood.

**Method:**

A mixed‐method approach was used including statistical analyses (*n* = 210) and interpretative phenomenological analysis (IPA).

**Results:**

It was shown that there was a significant difference between bereaved (*n* = 34) and nonbereaved (*n* = 176) respondents concerning, psychological distress, posttraumatic stress symptoms, and self‐rated health. Three themes were found by using the IPA approach (*n* = 9): Living in traumas, carrying heavy baggage, and living with change.

**Conclusion:**

The respondents described personal feelings of grief that are not expressed in their outward appearance or behavior in their daily living. When meeting young adults that have lost someone close in childhood or adolescence, this is important to have in mind.

## Abstract 2

To lose somebody close under traumatic circumstances during childhood or adolescence are heavy belongings. Still after as many years as eight or nine, this experience can be interpreted as living in the trauma, carrying heavy luggage, but anyhow living with change.

## 1 INTRODUCTION

To lose a person close suddenly, a parent, a sibling, a relative, or a friend, during childhood or adolescence can be devastating. Several studies concerning children and adolescents who have lost a parent have shown the negative impact of the loss, mental health problems such as depression, anxiety, posttraumatic stress, and drug abuse (Brent et al., 2012; Dopp & Cain, 2012; Dowdney, 2000; Dyregrov, 2010; Kirwin & Hamrin, 2005; Melhem et al., 2011, 2013; Nilsson & Ängarne‐Lindberg, 2016).

To, by death, lose a person close suddenly and unexpectedly and at the same time self‐having been in danger can be defined as a traumatic loss (Boelan, et al., 2017; Raphael et al., 2004). It is relatively common that death caused by natural disasters often are sudden, unexpected, and can include additional traumas such as facing life‐threatening situations and witnessing damaged corpses (Malone, 2016; Nakajima et al., 2012). Traumatic bereavement or traumatic losses are not seldom more stressful, complicated, and difficult to recover from than the bereavement of a natural death that was expected (Barlé et al., 2017; Malone, 2016). It has been documented that those traumatic experiences such as described earlier can interfere with the normal grief process (Barlé et al., 2017; Nader & Salloum, 2011). Grief can be seen as a normal and natural process after bereavement (Stroebe et al., 2007). Stroebe et al. (2008) defined grief as the emotional reaction to bereavement, incorporating diverse psychological and physical reactions.

According to Worden (2018), the grieving process includes four steps: To accept the reality of the loss, to process the pain of grief, to adjust to an environment where the deceased is missing, and to relocate the death in one's life and find ways to memorialize the deceased person. It has been found that adolescents have not yet developed social or emotional maturity to fully incorporate and process loss, grief, and trauma (Malone, 2016), something that has also been shown in Sweden (Bylund Grenklo, 2013; Dyregrov, 2007; Jacobsen, 2004).

The death of a loved one during adolescence can therefore destabilize important developmental issues such as: (a) trusting others and the world; (b) gaining a sense of belonging; and (c) gaining a sense of mastery (Balk, 2014).

Trauma‐related interference with the adaptive grieving processes can occur due to the traumatic aspects of the death. This may hinder or complicate the issues of grief including, the ability to recover from shock related to the loss, reminiscing, grief‐related dreaming, aspects of the relationship with the deceased, issues of identification, and the processing of anger and rage (Nader & Salloum, 2011).

Grief can be expressed differently, sharing emotions of loss with others or avoidance talking about it. Traumatic grief focuses on the traumatic circumstances of a death; the simultaneous occurrence of trauma and grief can strengthen the trauma symptoms (Nader & Salloum, 2011). This can in turn relate to increased demands psychologically and is a risk for psychological ill health (Dyregrov, 2010) and may, for some adolescents, lead to persistent complex bereavement disorder (APA, DSM‐5, 2013) or prolonged grief (WHO, ICD‐11, 2018) (Revet et al., 2020).

Children and adolescents are almost completely dependent on their parents, and a loss could lead to socioeconomic problems and posttraumatic stress symptoms, which in turn, can lead to impacts on personality (The Swedish Family Care Competence Center, 2014). Providing early prevention support programs for grieving children can help the young people adapt to their losses. Professionals who have responsibility need to possess knowledge about early intervention, development, and the forms that reactions can take (Nilsson & Ängarne‐Lindberg, 2016). Young people who are not supported in the early phases of grieving can develop serious emotional and behavioral problems after losing a parent (Kirwin & Hamrin, 2005). For young people, the sorrow and grieving take time, it is common with delayed mourning and reappeared sorrow (The National Board of Health and Welfare, 2012, 2013a,b). To lose somebody close can lead to loneliness, change of world view (Melhem et al., 2013), withdrawal from friends and activities (Dopp & Cain, 2012), problems in peer relations, and lower competence in work (Brent et al., 2012). Stikkelbroek et al. (2016) found that adolescents who had lost a sibling by death, including stepbrothers, stepsisters, half‐brothers, and half‐sisters, reported more internalizing problems after the loss, compared to the nonbereaved. No studies have been found that explore children´s or adolescents’ sudden loss of grandparents (PsychInfo, PubMed, and Scoupus Databases).

Many young people, between 10 and 15 years of age, from Sweden, were on holiday in Southeast Asia during the Christmas season in 2004 with their family when the tsunami came. A huge wave hit the coast, and many people struggled for their life. In total, 543 Swedish citizens died, some of them parents, grandparents, siblings, or friends. Some of them lost one, two, or more of their close ones during the tsunami. The survivors were able to leave the disaster‐struck areas and return to Sweden far from the area where they experienced the tsunami.

Studies have been performed directly after the tsunami in the affected countries in Southeast Asia and in Norway, on adults and young people after the disaster. One Norwegian study (Jensen et al., 2009) found that 3% of those who had lost somebody close had symptoms significantly associated with posttraumatic stress 10 months post disaster.

Nader and Salloum (2011) have stated that future prospective studies are needed that include children with different relationships with the deceased. Melhem et al. (2011) draw the conclusion that future studies need to examine the long‐term mental health and developmental outcomes, for children and adolescents losing a person close to them. There has been a lack of knowledge about how these children experienced loss of a person close to them many years later, in young adulthood. Revet and colleagues (2020) even posed the question if grief is a process that is limited in time. It can be of importance to describe what this group experiences and feels several years later about their loss. Also, no studies have been found that have investigated grief for children and adolescents in young adulthood, several years after the sudden death of a person close to them.

Therefore, one aim of this study was to explore how sudden loss during childhood or adolescence sustains long term into young adulthood after a natural disaster. Is there a difference between those who have not lost someone and the ones that have lost someone close regarding psychological distress, posttraumatic stress, and self‐rated health (SRH)?

A second aim was to explore how young adults have handled traumatic loss during childhood or beginning of adolescence eight or nine years post the disaster.

## 2 METHOD AND MATERIAL

The present study used a mixed‐method approach (Creswell, 2018) to describe and to get a deeper understanding of the long‐term effect of earlier loss of someone close. A mixed‐method approach takes advantage of using collection and analysis of quantitative data followed by a collection and analysis of qualitative data. This method gives the possibility to address research questions at different levels, and to explore phenomena, in this study the long‐term effects of earlier loss.

### 2.1 Participants

In 2004, 627 children, aged 10–15 years, living in Stockholm County, were registered by the police between December 27, 2004 and January 15, 2005, upon their return to Sweden from countries in Southeast Asia after the tsunami there. The reason to choose children and adolescents aged 10–15 was that they have reached a stage in their development where they could remember the natural disaster and could express their thoughts in words (Braun‐Lewensohn, 2015). All participants included in the main study were divided into four exposure groups (Adebäck et al., 2018). All members of exposure group four had been physically present on the beach or in the water or had seen the wave, had experienced a threat to their own lives and/or a threat to the life of a family member, separation from parents during the tsunami, and loss. Exposure groups one to three experienced one, two, or three of these exposures. The sample in this study is statistically selected from exposure group four.

### 2.2 Procedure

In August 2013, questionnaires together with information letter were sent to 627 young survivors of the tsunami in Southeast Asia. Of those 255 young adults (42%) answered the questionnaires, and 210 (82%) were in a place that was hit by the tsunami and were included in this study. Of these 210 young adults, 34 (16%) answered that they had lost someone close and 176 (84%) answered that they had not lost someone close. In the quantitative part of this study, the ones who had not lost anybody (176) were compared with the group (34) who had lost someone. The questionnaires included questions about loss. A close person was defined as a parent, a sibling, a grandparent, a friend, or a friend to the family. Due to the disaster, more than one person could be lost. Of those who had lost someone, a total of 34 young adults, 16 accepted to be interviewed and got an invitation to the interview. Nine persons (five women and four men) could be reached and accepted to be interviewed. The mean age of this group was 13 years in 2004 and 22 years at the time of the interview in 2014 and 2015 (range 19–25 years) (Table 1).

**TABLE 1:** Participants (*n* = 34) that have lost someone close to them

	Mother	Father	Sibling	Grandparent	Other relative	Friend	Boy‐ or girlfriend	Friend to the family
Lost	8	8	4	6	1	11	1	19

*Note*: Because of the fact that one person included in the study could have lost more than one person in the tsunami, the numbers of lost persons are not 34.

### 2.3 Questionnaires

#### 2.3.1 General health questionnaire (GHQ‐12)

The 12‐item GHQ‐12 was used to assess general psychological distress rated over the past few weeks (Goldberg & Williams, 1988; Sconfienza, 1998; Wahlström et al., 2008). Each item scored zero to three, giving a range of 0–12, and the higher the score the more psychological distress the respondent expressed. The response rate was 99%, one of the respondents not losing anyone close did not answer these questions. Cronbach's alpha for the present study group was 0.83–0.87.

#### 2.3.2 Impact of event scale‐Reversed (IES‐R)

The IES‐R is an often‐used scale identifying posttraumatic stress symptom, consists of 22 items, and was used (Sconfienza, 1998; Sveen et al., 2010; Wahlström et al., 2008). In this study, the degree of posttraumatic stress symptoms in the last week, eight years later, due to the tsunami 2004 were examined. Each item was rated on a five‐point scale and each answer ranged from 0 = not at all to 4 = extremely. The response rate was 99%, and one of the respondents not losing anyone close did not answer these questions. Cronbach's alpha for the present study group was 0.95.

#### 2.3.3 Self‐rated health

SRH is a commonly used research tool for the rating of subjective, self‐perceived health status. SRH is in the present study assessed by the question “How would you rate your general state of health?”. Responses were rated on a five‐point scale and each answer ranged from 1 = very good to 5 = very poor. The validity and reliability have in former studies been found to be good for this question (Nixon Andreasson, 2010). The response rate was high; 33 of the bereaved young adults answered this question and 174 of the nonbereaved.

### 2.4 Interview study

An information letter about the study was sent out including a telephone number where the participant had said they could be reached. If we had found no telephone number, the participant was instructed to contact us. The first author contacted the participants by phone and booked a time for interview. The semistructured interviews were all done by telephone by the first author and an interview guide was prepared for this study. The interviews lasted between 30 and 90 min. The interviewer started with a broad question as how you are right now, and simple questions followed as what your memories of the tsunami are. The participants had the possibility to choose an interview face‐to‐face but this alternative was not chosen by anyone. All interviews were recorded and transcribed verbatim.

### 2.5 Data analysis

The questionnaires were analyzed using *t*‐tests to examine differences between the group of 34 who had lost someone close in the 2004 tsunami, and the 175 respondents who had not lost someone close.

Interpretative phenomenological analysis (IPA) was used to examine experiences of losing someone close several years after the loss (Smith et al., 2012). The answers describe each person's unique experiences, as well as their common experiences. The researcher using IPA has an active role in the research process as interpreter of the participants’ experiences. The analysis of the interviews followed the rules and steps according to Smith et al. (2012) and the material was reread to gain an initial understanding of the participants’ experiences and worldviews. All three authors read through the texts and important themes were found, which resulted in a list that reflected the participants’ experiences first on a descriptive level and then on a more interpretative level. The themes were discussed by all the authors, and changes were made accordingly. The IPA procedures has also been followed when writing the manuscript.

### 2.6 Ethical consent

All young adults were informed verbally, and informed consent was received from all participants. The results are presented to guarantee anonymity and in form of deidentified data and group‐data only. The study was approved by the regional ethical review board in Stockholm, Sweden (Dnr:2013/619‐31/5).

## 3 RESULTS

### 3.1 Results from the questionnaires

There was a significant difference between bereaved young adults and nonbereaved young adults eight years post disaster. The results for GHQ‐12 (effect size 0.41), IES‐R (effect size 0.87), and SRH (effect size 0.65) were all statistically significant (Table 2).

**TABLE 2:** Comparison between the bereaved and nonbereaved

Mean			Standard deviation		
Outcome variable	Berevead *n* = 34 mean	Nonbereveaved *n* = 176 mean	Berevead *n *= 34	Nonbereaved *n* = 176	Effect size
GHQ‐12	24.7	22.7	5.7	4.8	0.41
IES‐R	60.1	43.0	22.0	19.2	0.87
Self‐rated health	2.2	1.7	0.8	0.9	0.65

The participants that had lost someone had more posttraumatic stress symptoms than the ones that had not loss someone close, according to IES‐R (Table 2). According to GHQ‐12 and SRH, the bereaved also had psychological distress and a lower SRH compared to the nonbereaved (Table 2).

### 3.2 Results from the interviews

The respondents managed the loss in different ways and had thoughts about how it had changed their lives. The feelings they expressed have been interpreted and sorted into three themes: *Living in traumas, carrying heavy baggage, and living with change*.

### 3.3 Living in traumas

According to the participants, it was devastating having experienced a natural disaster like the tsunami 2004. To be exposed to this was something awful to memorize. It was described as a terrifying episode in life. The experience was frightening, and they never wanted to be part of this again.

…the tsunami was really an experience that was…, you saw things, you experienced things that nobody would ever want to experience.

These children and adolescents had experienced the natural disaster but also loss of persons close to them. They had lost a person such as one or two parents, a sibling, a grandparent, or a friend.

I think about this every day. I think about my parents every day and so.

They described the situation they had been in as horrible and the loss of someone close made everything even worse. It was described as carrying a double burden and something painful and difficult to manage. The participants described it as something difficult to understand; the whole situation and their experiences were mixed up with the loss of someone close. It was difficult to sort things out; they had two different memories they wanted to separate but could not because all their memories and emotions were mixed. The important element from the interviews was, however, the loss.

One tries to separate…there are two different memories that I do not want to put together. Today everything is just a mass of sorrow in my body.

However, despite this awful situation as described by the participants, and the fact that they had lost someone close to them, many of them thought that they were fortunate in some way compared with others who had lost someone. They experienced that they, for example, had good self‐confidence, a nice family, good friends, and possibilities to have jobs or studies. Many of the interviewed tried to think positively; this was not easy, but they really tried.

When I have bad thoughts, I try to transform them instead into good thoughts.

The participants described that other people who have not had these experiences could complain over things that they thought was nothing to complain about. Some of them had an experience of other young people who were depressed, self‐hurting, and had eating disorders. They described that they wanted to live their lives as well as possible, they thought they were lucky to be alive, and did not want to be negative because they had survived something horrible.

The participants could ask themselves why just they had survived the tsunami 2004. In some cases, this could lead to feelings of guilt, in some cases very much guilt, bad conscience that they had survived but not others. At the same time, thoughts about what could have happened were frightening. However, the participants talked about how proud the person that died should be over them. You can say that the bad conscience had been taken care of through pride.

Sometimes I can feel that it might just as well have been me…it could have been just as well me who could have died over there.

### 3.4 Carry heavy baggage

After nine years, participants have had time to mourn. During their passage through young adulthood, they did carry belongings consisting of sorrow and sadness, which sometimes was very heavy. However, even if these feelings have changed over time, and they said they had good health now. Moments of bad feelings were common. When they felt bad, a sadness could come over them in different situations and sometimes these moments were very painful.

And yes, “everyone bears some kind of baggage, and this is the heaviest load I have borne. Sometimes it is a little more difficult to forget but on many other days the burden is not that great.”

The participants found that they had learned to manage their sorrow during the years when becoming young adults. The feelings could be awakened by different situations and this experience was highly individual. By listening to a song or looking at a picture, they allowed themselves self to be sad and to mourn. To avoid thinking about what they experienced, they use strategies such as studying, working, or using internet.

Christmas, the time when the disaster happened, was the special days that could be hard every year. Other hard days were the birthdays of those whom they had lost. These were days that could be devastating or even got them to feel like panic.

Their posttraumatic stress symptoms arose when they got reminders of the tsunami. One kind of reminder occurred when someone mentioned the tsunami and what happened or when the tsunami was mentioned suddenly and unexpectedly. The whole situation came closer when they got a question about it. A question raised could be about how many you are in the family. Even other questions woke up feelings of the loss and these questions could be hard to answer. The whole situation could be revived when they got a question about their experience.

When someone unexpectedly mentions the tsunami to me…I experience panic in principle…panic driven anxiety, I cannot manage anything just then, what I do, say, or think.

The respondents described posttraumatic stress symptoms, and some had also been formally diagnosed, by a medical doctor, with posttraumatic stress disorder, PTSD. They had been diagnosed with PTSD long after first experiencing symptoms. Posttraumatic stress symptoms could make it difficult for them to concentrate and they might recapture things from the disaster even if they did not want to. They described sleeping problems or physical reactions like sweating, breathing problems, and whimpering.

They often had to rely on themselves, and they were often alone when feeling sad and therefore had to manage the sorrow by themselves. However, it was important to have somebody that could comfort them and listen to them and talk about what were their experiences during the tsunami. Some were helped by having someone who could give them support when it was needed. A person, who could let them feel and someone to talk about the situation with. Often, it was difficult for people who had never experienced what they had experienced to understand their feelings and that they could have died. If you meet someone with the same experiences, this person could understand what they had been through and talk about it. On the other hand, maybe, these persons would not have talked about their experiences because of respect for the other person. The participants thought that if they had talked about the loss for example, with a parent, it could have prevented ill health.

In the past years as soon as anyone took up the tsunami or…it is understandable that he or she felt so bad about this that she became anxious.

The participants said that they had not been able to handle the sorrow in a good way and the tsunami experience could still be hard to talk about. Others avoided the whole situation and thought very little about what happened. They did other things because of the unthinkable, what happened during the tsunami, as a way to stay away from the sorrow. Sometimes it was good for them to avoid thinking and sometimes it had been bad for them to avoid things. As one example of a bad thing, some of them talked about how they might be overwhelmed by all the things that they had avoided. Others said that they even used drugs to avoid thinking.

### 3.5 Living with change

It was found that the disaster had changed the lives of the participants in many ways. But some of them said that they did not know if this experience had changed them at all. They did not know how they might have been in the present if they had not lost someone close to them. A change that was obvious was that they could think more of the consequences of what they had done and what this could lead to. They described those experiences of the tsunami changed them so that today they were more stable or felt that there was stronger unity in the family than before. However, they had become more anxious, they had a harder time with stressful incidents, that they could put themselves down or that they could become furious rather quickly. In summary, they thought a lot ahead of things and they thought that they are now different from what they once were as a person.

I can have thoughts, I am perhaps more careful, that I think that it can perhaps happen. I think more about consequences.

Returning to the place where they had been during the disaster was important. While there, they got the opportunity to adapt in some way. However, being there could made them feel bad, but they could meet their own fear, which also had some positive effects.

I can say that it has helped to be able to rework everything and to return there… And I feel that when I am there, I can meet my fears and overcome them and that feels good. Maybe, it is not always good to be there, but it can sometimes be.

They described that they had difficulties with relationships after living through the 2004 tsunami and their losses. At the same time, some of the participants said that they did not know who they could rely on. Specifically, the impact of disaster had also influenced the way the family kept together.

During the years post disaster, these children and adolescents had, under many years, thought about their loss and believed that they had not functioned like others of their age.

It feels really bad that we have lost our whole teenage years just by feeling depressed and not being able to have fun and to just let go. I have gone around and been unable to stop thinking, over and over again about the past. I think all my teenage years have just been thrown away. That I just could not enjoy and have fun.

Most of the views were that even if it is going to be hard sometimes, the participants had a positive view of the future. In the future, it was important to remember the person who had died. They could think about the life course of that person and not that they died.

No, but I think I will manage pretty well, and it is obvious that it will be a little hard on the anniversary… the day to remember and on holidays, is always somewhat difficult. But I keep learning to handle all that better and better.

## 4 DISCUSSION

The main result from this study was that after eight‐ to nine‐years posttraumatic disaster, the participants were still influenced by the traumatic loss. The bereaved young adults had significantly higher scores for general psychological distress, posttraumatic stress symptoms, and had lower SRH compared to the nonbereaved. With respect to the *p*‐value and effect size, the consequences from the results from GHQ‐12 and SRH were not as substantial as the posttraumatic stress symptoms. As we have found no other study that has followed young people up to nine years after a natural disaster nor any study investigating both loss and trauma statistically after so many years, it is not possible to make comparisons. However, to be noted are that the traumatic loss added to the traumatic experience of tsunami and that they showed significantly higher levels of posttraumatic stress symptoms.

The results from the IPA gave three themes: *Living in traumas, carrying heavy baggage, and living with change*. There were also found inner feelings of mourning and that can be called traumatic grief. The participants described feelings of trauma and sorrow but also positive feelings and pride. They felt that living with the trauma and living with the loss was very hard for them, which has also been reported by Nader and Salloum (2011). From the interviews it was obvious that the respondents still struggled with feelings of trauma and loss. Something which makes the proposed question about if the grieving process can be said to be limited in time, very relevant (Revet et al., 2020).

Nevertheless, it seems as if the respondents in this study had been able to go through the steps in the grief process outlined by Worden (2018) even if there had been difficulties on the road in doing so. From the theme *living in trauma* it could be understood that the traumatic death and loss made it more difficult accept the reality of the loss.

Traumatic death had been something hard to accept according to the respondents in the present study and they had mixed feelings; the tsunami was by itself a horrible experience, but these children and adolescents had also lost someone close to them, which made it worse. So, the posttraumatic symptoms interfered with the grief process. The theme, *carrying heavy baggage* probably made it more difficult to go further and process the pain of grief. One could imagine that the *heavy baggage* with the grief and sorrow was so heavy that the process was extra demanding.

However, our respondents’ third theme *living in change* showed that the grief process was going on and they had managed to pass both passages three and four described by Worden (2018). Concerning to adjust to an environment where the deceased is missing and to relocate the death in one's life and find ways to memorialize the deceased person, the respondents in this study described themselves as having a life situation characterized by doing well in society but with inner feelings of loss. Some said that they thought about that the deceased would have felt pride over them if they had seen them today. The theme *living in change* has been noted in some other studies on grieving adolescents, for example. in a study by Amstrong and Shakespear‐Finch (2011) who found that bereaved adolescents reported higher posttraumatic growth in the domain called new possibilities and personal strengths. Yet another study, even if the death was in cancer and maybe not sudden, indicated that bereaved adolescents reported that their parent's death represented great changes for them in that they no longer had that parent to talk to, and in addition, as adolescents had to try to cope with difficult situations by themselves (Delihn & Reg, 2009).

Still, it was hard to see on the outside that these young adults were not feeling so well. The present study showed that the internalizing difficulties were existing even after a long time. The present results are also in line with the results from Stikkelbroek et al. (2016) who concluded that internalizing problems increase more within 2 years in adolescence after family bereavement in comparison to the nonbereaved.

Blank and Werner‐Lin (2011) found that the relationship between a child and parent is complex and dynamic, changing as the child grows older and begins to see the parent as a multifaceted person. They suggested that children revisit and reintegrate the loss of a parent as their emotional, moral, and cognitive capacities mature and as normative egocentrism and magical thinking decline. Maybe this underlines that the respondents in our study were still working with their loss and had feelings of sorrow.

Another study by Hamdan et al. (2012) found no statistically significant difference in health risk behaviors, during a 3‐year period after a parent´s death, which was shown also in this study. Only occasionally, the inner problems in the present study were followed by behavior that is not so well seen in our society, for example excessive drinking. In yet another study, an increased risk for psychiatric care was found for women who had experienced a loss of a sibling during adolescence (Rostila et al., 2019). In that study, they speculated that family‐related psychosocial conditions shared by siblings in childhood might account for the association between sibling death and psychiatric care in adulthood. When siblings were said to be important for the interviewed in the present study, maybe these associations could have been present in our study too.

In the present study, it was found that the respondents needed support for many years after the event and some of them still wanted professional help. The Swedish social services agency, the National Board of Health and Welfare (2013a,b) states that the child needs security, shelter, calming, a feeling of belonging, and hope and assurance from the social network and society. This is in line with what Nader and Salloum (2011) found, that among the protective factors for complicated grief are parenting, home conditions, social support, nature of attachments, and other support systems. Bereaved families would benefit from a knowledgeable follow‐up contact with professionals in weeks and months after the death to offer condolences, answer questions about the death, and provide referral to grief resources in the community (Mayer, 2017). The results from the present study show that children and adolescents need follow‐up contact for a longer time than just a few months after a natural disaster. Some of our respondents talked about how they wanted support in young adulthood from a professional, individually or in group. It was also found that many managed by themselves after the natural disaster and we can wonder if the support system and the home conditions, acute and long term, were good enough. How much support children or adolescents need after a natural disaster is also a question to be answered? In a study by Rosner et al. (2010), it was found that interventions for symptomatic or impaired children and adolescents tended to show larger effect sizes than interventions for bereaved participants without symptoms. Some of the respondents in this interview study showed more difficulties and maybe it is the ones that would be better helped by interventions, even as adults.

A study by Mayer (2017) found that surviving family members frequently struggle with multiple questions and may blame themselves or others for not preventing the death. In the present study, it was also found that feelings of guilt over surviving were common in children and adolescents who were close to someone who had died.

### 4.1 Limitations

The response rate was only 32%, which can be a limitation of the present study. No dropout analysis was made because of the terms of confidentiality. However, we can only speculate that there were several who did not answer because they had not been in an area hit by the tsunami, a phenomenon that has been found in a survey study in Norway (Hussain et al., 2013). Another limitation with this study was the possibility of recall bias regarding what happened eight years ago. The possibility that the most affected children or adolescents may have chosen not to answer the questionnaire and therefore were not a part of the study could also be a limitation. A limitation with the qualitative part was the small sample. We could not reach all participants and they became the seven dropouts in the study. Of course, it is different if you lose a parent, or a sibling compared with if you lose a friend to the family. All participants had lost a parent, a sibling, a grandparent, or a friend. Maybe we should have left out friends to the family in this study to make the study more stringent. In this study, we do not know if they have received any help after the loss. Some of the participants mentioned that they have gotten help in some form, a professional speaking person or some medical help.

### 4.2 Strengths

A strength of this study was our ability to use the police register to find the respondents, a possibility in the present study that is unusual. Our study group consisted of individuals who were 10‐ to 15‐years‐old at the time of the disaster – a stage in their development that enabled them to remember and to explain their experiences (Braun‐Lewensohn, 2015). Other strengths were that many young adults responded, in spite of the difficulties reported in earlier studies with reaching persons of these ages, especially males. A strength was also that the questionnaire could be completed over the internet no matter where the respondents were living. Every question in the questionnaire had the alternative “do not remember,” a strength when only an alternative was used by a small group of this study group. The participants were also very interested; they remembered much of what had happened to them and they talked easily and at some length. In this study, the impact of sudden loss after a natural disaster was examined, something that also can be true after a car crash, robbery, and other potential psychological traumas. In that case, we must meet children and adolescents, after they had gone through different traumas, in a much better way and for a longer period of time than mostly is done today.

## 5 CONCLUSION

To lose somebody close under traumatic circumstances during childhood or adolescence are heavy belongings. Still after as many years as eight or nine, this experience can be interpreted as living in the trauma, carrying heavy baggage, but anyhow living with change. With much struggle and it is not always shown, the young adult can be doing well in the society, but it is not uncommon that they are troubled by inner feelings of loss and posttraumatic symptoms.

## CONFLICT OF INTEREST

There is no conflict of interest.

### PEER REVIEW

The peer review history for this article is available at https://publons.com/publon/10.1002/brb3.2563

## Data Availability

The data that support the findings of this study are available on request from the corresponding author. The data are not publicly available due to privacy or ethical restrictions.
